# Molecular Basis for Actin Polymerization Kinetics Modulated by Solution Crowding

**DOI:** 10.3390/biom13050786

**Published:** 2023-05-02

**Authors:** Bryan Demosthene, Myeongsang Lee, Ryan R. Marracino, James B. Heidings, Ellen Hyeran Kang

**Affiliations:** 1 NanoScience Technology Center, University of Central Florida, Orlando, FL 32826, USA; 2 Burnett School of Biomedical Sciences, University of Central Florida, Orlando, FL 32827, USA; 3 Department of Physics, University of Central Florida, Orlando, FL 32816, USA; 4 Department of Materials Science and Engineering, University of Central Florida, Orlando, FL 32816, USA

**Keywords:** actin assembly, solution crowding, filament elongation rate, molecular dynamics, diffusion coefficient

## Abstract

Actin polymerization drives cell movement and provides cells with structural integrity. Intracellular environments contain high concentrations of solutes, including organic compounds, macromolecules, and proteins. Macromolecular crowding has been shown to affect actin filament stability and bulk polymerization kinetics. However, the molecular mechanisms behind how crowding influences individual actin filament assembly are not well understood. In this study, we investigated how crowding modulates filament assembly kinetics using total internal reflection fluorescence (TIRF) microscopy imaging and pyrene fluorescence assays. The elongation rates of individual actin filaments analyzed from TIRF imaging depended on the type of crowding agent (polyethylene glycol, bovine serum albumin, and sucrose) as well as their concentrations. Further, we utilized all-atom molecular dynamics (MD) simulations to evaluate the effects of crowding molecules on the diffusion of actin monomers during filament assembly. Taken together, our data suggest that solution crowding can regulate actin assembly kinetics at the molecular level.

## 1. Introduction

Actin filament assembly dynamics drive various cellular processes, including cell motility, cell division, intracellular transport, and cell shape changes [1,2,3]. The assembly of actin monomers into filaments is tightly regulated by numerous actin-binding proteins and various intracellular environmental factors [3,4]. The intracellular environment is crowded with diverse molecules, such as ions, nucleic acids, proteins, and macromolecules. Macromolecular crowding leads to the excluded volume effects and depletion forces that affect in vivo biochemical reactions and biomolecular interactions [5,6,7]. The effects of solution crowding include influencing protein stability and protein–protein interactions, altering protein conformation and structure [8,9,10,11], and influencing protein assembly and reaction rates [9,12,13].

Macromolecular crowding affects actin polymerization kinetics [14,15,16,17,18,19], the stability of actin monomers and filaments [18,19,20], and filament bending stiffness [21]. The critical concentrations of actin decrease [14,18,20,22], and actin polymerization is enhanced [14,16], in the presence of polymeric crowding agents, such as polyethylene glycol (PEG) and Ficoll [20]. Depletion interactions raised by these inert polymeric crowders have been shown to induce actin bundling [23]. In contrast, protein crowders, such as bovine serum albumin (BSA) and lysozyme, can contribute enthalpic effects that may inhibit actin polymerization for oppositely charged proteins [15]. It has been suggested that the excluded volume effects raised by macromolecular crowding can influence filament self-assembly [10,15,16]. However, recent studies have shown conflicting results of protein interactions and assembly in the presence of different types of crowding agents [15,17,24]. While the effects of macromolecular crowding on bulk actin polymerization have been studied, the mechanisms behind how solution crowding influences actin polymerization kinetics at the molecular level are not fully understood.

Here, we investigated how solution crowding modulates actin filament assembly kinetics through combined experimental and computational approaches. We determined the effects of solution crowding on the elongation rates of individual actin filaments utilizing real-time total internal reflection fluorescence (TIRF) microscopy imaging with a functionalized flow cell. All-atom molecular dynamics (MD) simulations demonstrated that actin monomer diffusion near the barbed ends of filaments, as well as filament electrostatic energy, depend on the type of crowding molecules, thereby influencing actin polymerization. The relevance of our findings and the potential molecular mechanisms for actin assembly in crowded environments are discussed.

## 2. Materials and Methods

### 2.1. Protein Purification and Sample Preparations

Actin was purified from rabbit skeletal muscle acetone powder (Pel-Freeze Biologicals Inc., Rogers, AR, USA) and gel-filtered over a Sephacryl S-300 column equilibrated in buffer A (2 mM Tris-HCl, pH 8.0, 0.2 mM CaCl_2_, 1 mM NaN_3_, 0.2 mM ATP, and 0.5 mM DTT), as previously described [25]. Actin monomers were fluorescently labeled with Alexa 488, as previously described [26]. Biotin- and pyrene-labeled actin proteins (>99% purity) were purchased from Cytoskeleton, Inc. (Denver, CO) and reconstituted in buffer A. Prior to each experiment, actin monomers were centrifuged at 60,000 rpm at 4 °C for 1 h using a Sorvall MTX 150 ultracentrifuge (Thermo Fisher Scientific, Waltham, MA, USA) in an s100-AT3-2029 rotor (Thermo Fisher Scientific, Waltham, MA, USA) to remove aggregates. Cation exchange was performed on calcium-bound actin monomer (G-actin) to replace Ca^2+^ with Mg^2+^, equal to the concentration of G-actin plus 10 µM and 0.2 mM EGTA. 

Stock buffers containing crowding agents were prepared at 15% *w*/*w* PEG MW 8000 (~18.8 mM), 30% *w*/*w* BSA (~4.5 mM), and 40% *w*/*w* sucrose (~1.2 M). After addition of the crowding agent, buffer A was used to bring the weight up to the final solution weights of 10–15 g. Actin (Alexa- or pyrene-labeled) was polymerized by the addition of 1/10th volume 10× polymerization buffer (100 mM imidazole, pH 7.0, 500 mM KCl, 20 mM MgCl_2_, 10 mM ATP, and 10 mM DTT) at room temperature for 1–2 h. 

### 2.2. Flow Cell Preparation

**Figure 1:**
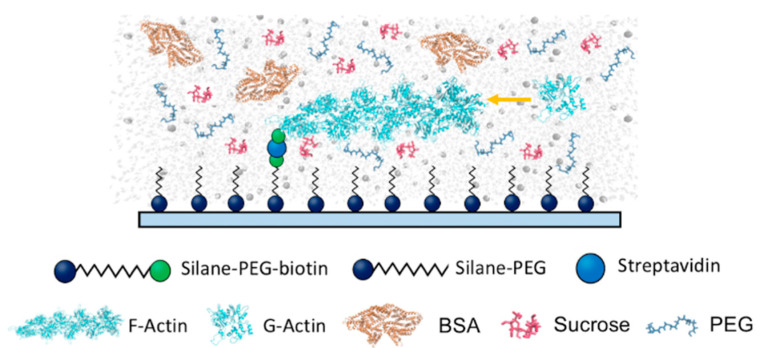
Schematic diagram of a functionalized flow cell designed to monitor actin filament assembly in the presence of various crowding molecules. Actin monomers (biotin-labeled) are anchored on a substrate functionalized with silane-PEG-biotin and streptavidin. Actin filament assembly is initiated by the addition of polymerization buffer into the flow cell and monitored using TIRF microscopy.

A functionalized flow cell was prepared following a modified protocol, as previously described [27,28,29]. A schematic diagram of the functionalized flow cell for observing filament assembly in the presence of solution crowding is shown in Figure 1. The coverslips were sonicated in 1 M KOH, followed by 1 M HCl, and finally 70% ethanol for 45 min at 60 °C. Coverslips were thoroughly rinsed with ddH_2_O at 60 °C after each sonication step. Silane-PEG (MW 2000) and silane-PEG-biotin (MW 3400) were purchased from Laysan Bio, Inc. (Arab, AL, USA), and 1 mg/mL stock solutions of silane-PEG and silane-PEG-biotin were prepared in 80% ethanol and 1% 1 M HCl solution. The stock solutions were mixed at a ratio of 1:350 between silane-PEG-biotin and silane-PEG. The functionalized coverslips were incubated overnight at 60 °C in the previously mentioned solution, then rinsed with 60 °C ddH_2_O, dried in a nitrogen stream, and stored in a dark, sealed container at 4 °C until needed. A flow cell was prepared by placing two strips of double-sided tape lengthwise along the coverslips. A clean microscope slide was placed onto the double-sided tape, adhering it perpendicular to the coverslip. Streptavidin solution (100 nM streptavidin and 5 mg/mL BSA in 1× KMI buffer) was loaded onto the flow cell and allowed to incubate for 5 min prior to the addition of actin samples.

### 2.3. TIRF Microscopy Imaging and Data Analysis

Mg-ATP actin monomers (1 μM, 20% Alexa-labeled, and 0.38% biotin-labeled) were flowed into the functionalized flow cell. Then, filament assembly was initiated by the addition of polymerization buffer containing various concentrations of crowding agents (PEG, BSA, or sucrose) at room temperature. To minimize photobleaching, the polymerization buffer was supplemented with 0.2 mg/mL glucose oxidase, 1 mg/mL catalase, and 15 mM glucose. Time-lapse images of filament assembly were recorded as soon as 1 min after initial polymerization with a Nikon Eclipse Ti TIRF microscope equipped with a Hamamatsu ORCA-Flash 4.0 C13440 digital camera, a 100× oil immersion objective (N.A. 1.49) with a pixel size of 0.07 µm/pixel, and a Nikon LU-N4 laser [21,30]. This allowed the observation of filaments from the time they were submicrons in length. TIRF microscopy images were taken every second up to 15 min and compiled into a time-lapse series using Nikon imaging software Elements (version 4.50).

ImageJ software (NIH) was used to generate kymographs from individual filament timelapses to demonstrate the change in filament lengths over time. Filament lengths were determined after image processing using the NIH’s ImageJ and analysis using Persistence software [31]. The changes in actin filament lengths of individual filaments under various crowded conditions were determined after analysis using OriginLab 8.5 software and compared to linear fittings of the kymographs. Filament elongation rates were measured, and statistical analysis was performed using one-way ANOVA, followed by post-hoc Tukey tests. 

### 2.4. Pyrene Fluorescence Spectroscopy

To measure bulk actin polymerization in crowded conditions, pyrene-labeled actin monomers (3 µM, 30% labeled) were polymerized by the addition of 1/10th volume 10× polymerization buffer [32] containing various concentrations of crowders (PEG, BSA, or sucrose). Baseline correction was applied based on the average fluorescence intensities for each sample over 5 min prior to the initiation of polymerization. The time course of pyrene fluorescence intensities was monitored using a SpectraMax Gemini XPS microplate reader with Molecular Devices SoftMax Pro software (version 7.0) (λ_ex_ = 365 nm and λ_em_ = 407 nm) for 2 h (Δt = 10 s) at room temperature (25 °C). The relative filament assembly rate was calculated by finding the time point at which the sample reached half maximum fluorescence intensity and then obtaining the slope of the line formed from the 10 fluorescence data points on either side of the half max point modified from a protocol by Doolittle et al. [33]. All slope values were normalized to the control value (buffer without crowding molecules).

### 2.5. Molecular Dynamics (MD) Simulations

All-atom molecular dynamics (MD) simulations were performed to evaluate filament assembly kinetics. We used molecular models for actin monomers and filaments (PDB ID: 3J8I) [34]. To efficiently simulate the filament assembly process, we simplified the polymerization by evaluating the association of a monomer located near the barbed end of the filament. We placed a preformed filament at the center of a given system and an actin subunit near the filament barbed end with an offset distance of 15 Å along the filament axis. An equilibrated filament model consisting of 5 subunits was used [21]. Here, G-actin was energy minimized and equilibrated for 5 ns. PEG (M_w_ = 385.25 g/mol), sucrose (M_w_ = 389.52 g/mol), or BSA (M_w_ = 66,540.3 g/mol) (PDB ID: 4F5S) molecules were considered as crowders. To mimic the experimental crowded conditions, we randomly solvated 15 PEG and 30 sucrose molecules near the filament and monomer complexes based on our previous study [35]. The concentrations of crowders (*C*) in the simulation were calculated to be *C*_PEG_ = ~6.5 mM, *C*_sucrose_ = ~13 mM, and *C*_BSA_ = ~0.4 mM. To solvate the crowders, Packmol was employed [36] to emulate with minimum 2.5 Å distance constraints on the actin filament and monomer complexes. In the case of BSA, two molecules were used due to BSA’s relatively large size compared to actin filaments. The complexes containing both actin monomers and filaments with or without crowders were solvated in a TIP3P water box under 15 Å of water padding conditions. To neutralize the system and mimic experimental conditions, 2 mM MgCl_2_ was solvated near the complexes.

Each system was energy minimized and thermally heated from 0 K to 300 K to remove any bad contacts and sudden movement of actin monomers. The equilibrium simulations were performed for 5 ns using NAMD version 2.12 [37]. CHARMM27 forcefield, including CMAP correlations for proteins, CHARMM35 forcefield for ethers [38], and CHARMM36 forcefield for sucrose [39], were applied for energy minimization, thermal heating simulation, and equilibrium MD simulations. To quantify the interactions between monomers and filaments, we applied particle mesh Ewald (PME) for long-range electrostatic energy interactions. Equilibrium MD simulations were carried out with consideration of the NPT ensemble (constant number of atoms, *p* = 1 bar and T = 300 K) and a time step of 2 fs.

### 2.6. Actin Subunit Diffusion Analysis from MD Simulations

To estimate the actin assembly kinetics from MD simulations, we calculated the mean square displacement (MSD) and diffusion coefficient of actin subunits located near the barbed end of the preformed filaments. The MSD of a subunit is defined as <$∆r_{i}^{2}\left(t\right)$>, which follows in accordance with Equation (1):

(1) $$<∆r_{i}^{2}\left(t\right)>=<{\left|r_{i}\left(t\right)-r_{i}(0)\right|}^{2}>$$

where $r_{i}\left(t\right)$ and $r_{i}(0)$ are atomistic coordinates of C_α_ atoms from actin residues at specific time trajectories and initial points, respectively. The time-dependent diffusion coefficient (*D*) was calculated from the MSD based on the Stokes–Einstein relation, as described in Equation (2):

(2) $$D=\frac{<∆r_{i}^{2}\left(t\right)>}{6t_{s}}$$

where *t_s_* is the applied time step during the MD simulations. 

To support the MSD values of the actin subunits, the inter-displacement differences (Δ*L*) between the filaments and subunits were calculated in accordance with Equation (3):

(3) $$\Delta L\left(t\right)=l\left(t\right)-l(0)$$

where l(*t*) is the time-dependent distance between the center of mass of the filament barbed end and that of a subunit, and l(0) is the initial length between the barbed end of the filament and subunit.

### 2.7. Polar Solvation Energy Calculation from MD Simulations

We measured long-range electrostatic interactions between actin filaments and subunits through polar solvation energy calculations. Due to the high polarity of actin, calculating the polar solvation energy is a useful approach for evaluating actin assembly. The polar solvation energies between the actin proteins and solvents were calculated based on the Poisson–Boltzmann equation. The polar solvation energies of the actin filaments and subunits were measured using Delphi v.4 with CHARMM forcefield [40]. We calculated two types of free polar solvation energies, such as ΔG, to see the dynamic characteristics of actin subunits and effect of molecular crowders. The solvation free energy ΔG was assessed using Equation (4):

(4) $$\Delta G=<E_{C}>-(<E_{F}>+<E_{G}>)$$

where *E_C_*, *E_F_*, and *E_G_* are the electrostatic energy of the filament and subunit complexes, filaments alone, and actin subunits alone, respectively.

## 3. Results and Discussion

### 3.1. Crowding Modulates Actin Filament Assembly Kinetics

**Figure 2:**
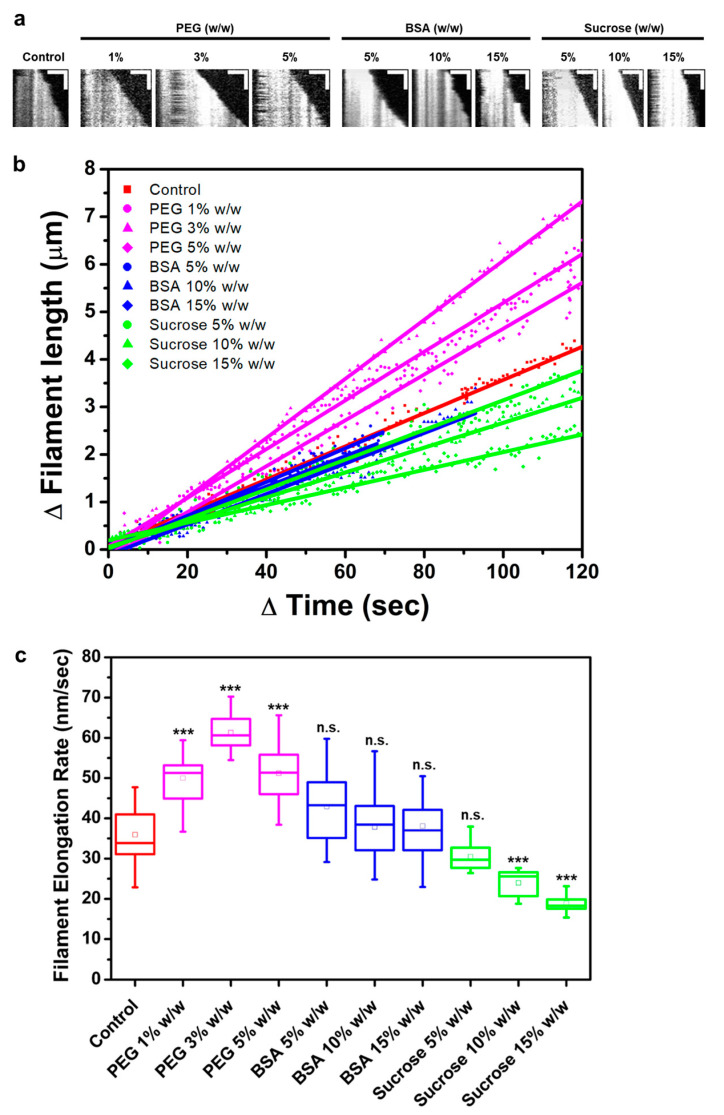
Direct visualization of individual filament assembly in crowded conditions using TIRF microscopy imaging. (**a**) Kymographs of representative filaments in each of the crowded conditions: polyethylene glycol (PEG) 1%, 3%, or 5% *w*/*w*; bovine serum albumin (BSA) 5%, 10%, or 15% *w*/*w*; and sucrose 5%, 10%, or 15% *w*/*w*. Horizontal scale bars, 3 μm; vertical scale bars, 20 sec. Changes in actin filament lengths over time. (**b**) Representative filament elongation while polymerizing in the presence of solution crowding shown in (**a**). Solid lines represent the best linear fits for each condition. (**c**) Box and whisker plots of actin filament assembly (growth) rates in the various crowded conditions shown in (**a**). The box represents the 10–90th percentile, the whiskers indicate standard deviation (S.D.), and the small box in the middle represents the mean. Tukey’s test indicated significant differences for all the conditions compared to the control (*** *p* ≤ 0.001; n.s. not significant). Number of filaments analyzed per each condition: 15 < *N* < 32.

We directly visualized the real-time elongation of individual actin filaments on functionalized flow cells using TIRF microscopy in dilute buffer and buffer solutions containing crowding agents. We chose PEG 8000 (herein PEG), BSA, and sucrose as crowding agents because they are biologically compatible molecules that can mimic some of the different types of molecules present within the cell. The concentration ranges used were similar to those of physiological conditions (up to ~40% of the total cell volume) [5]. High concentrations of crowding agents, including PEG, have been shown to induce bundling of actin filaments [4,23]; therefore, the concentration range of crowding agents in our experiments was kept below the bundle-inducing concentration. Based on the time-lapse TIRF images (Supplementary Videos S1–S10), we generated kymographs of individual actin filaments present in various crowded conditions (Figure 2a). Filaments grew steadily after the start of polymerization up to approximately 7 min, with one filament end growing faster than the other end. The fast-growing end to which monomers were constantly being added can be assumed to be the filament barbed end [41]. Changes in filament lengths over time were measured and plotted under various crowding conditions (Figure 2b). Filament elongation was analyzed until growing filaments did not result in significant overlap with other neighboring filaments within a given frame (between 60 and 120 s). This analysis allowed for measuring the average filament elongation rate per each condition.

The elongation rates of filaments depended on the type as well as the concentrations of crowding molecules (Figure 2c and Supplementary Table S1). Actin filaments elongated at an average rate of 35.9 ± 1.2 nm/s (~13.67 subunits/s) (±standard error of mean, S.E.M.) in dilute buffer conditions. This value is higher than previously reported rates for similar conditions (10 subunits/s for a similar actin concentration) [42,43]; however, the increase in our samples was consistent across all samples tested. In the presence of PEG (1%, 3%, and 5% *w*/*w*), the average filament growth rates were significantly increased, up to 61.3 ± 1.5 nm/sec (*p* ≤ 0.001), leading up to a 1.7-fold increase in rates with respect to the control. The addition of BSA did not affect filament elongation rates significantly, resulting in similar values compared with the control. In contrast, sucrose slowed filament elongation in a concentration-dependent manner (30.5 ± 0.8 nm/s for 5% *w*/*w*, 24.0 ± 0.8 nm/s for 10% *w*/*w* sucrose, and 18.9 ± 0.6 nm/s for 15% *w*/*w* sucrose).

**Figure 3:**
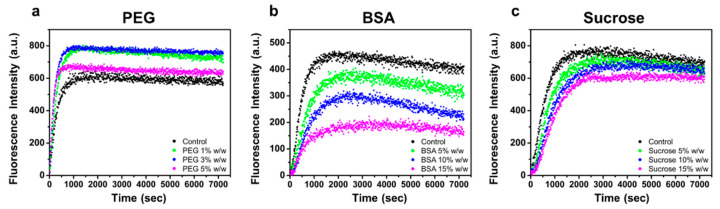
Pyrene assay analysis of bulk actin polymerization kinetics with solution crowding. Actin assembly measured through fluorescence intensity of pyrene-labeled actin (3 µM) across 2 h without crowding (control) or in the presence of (**a**) PEG (1%, 3%, and 5% *w*/*w*), (**b**) BSA (5%, 10%, and 15% *w*/*w*), and (**c**) sucrose (5%, 10%, and 15% *w*/*w*).

To test the effects of crowding on actin polymerization kinetics, we conducted bulk pyrene fluorescence assays. Actin polymerization rates can be estimated from the slope of fluorescence intensity at half-maximal polymerization (Figure 3) [33]. Based on this analysis, we estimated the relative rates of filament assembly (normalized to the assembly rate of the control sample) under crowded conditions (Supplementary Table S1). PEG induced faster actin polymerization (up to 1.4-fold increase), and sucrose decreased filament assembly rates (up to ~30% decrease) compared to dilute conditions, which is consistent with the filament elongation rates analyzed by TIRF imaging. In the case of BSA, the actin polymerization rate decreased from 89% to 46% of control with the highest concentration of BSA tested, contrary to our TIRF results in which no significant change was observed. 

Analysis of actin assembly kinetics observed through TIRF imaging and pyrene assays demonstrated how various crowding agents affect actin polymerization differently. The presence of the polymeric crowder PEG was shown to increase actin assembly in both methods, while the opposite was observed with sucrose crowding, where both methods exhibited a decrease in actin assembly kinetics. The difference in actin polymerization kinetics under these two conditions may be attributed to differences in their excluded volume, stabilizing effect by PEG [10,44,45,46], or due to nonspecific interactions between the crowders and actin [24,47]. The presence of BSA crowding decreased actin’s bulk assembly kinetics, whereas individual actin filament elongation rate measured from TIRF imaging was not significantly impacted by BSA when compared to control. This may be in part due to destabilizing effect by protein crowding through nonspecific interactions [48,49]. BSA’s electro-viscous properties at higher concentrations [50] may also contribute to the observed difference. 

The intracellular environment is a complex and dynamic system that contains a mixture of multiple different molecules and ions that influence biological interactions [4]. The minimal system in this study used polymeric crowder, protein crowder, and small molecule saccharide to emulate crowded conditions in the cytoplasm, providing an understanding of how solution crowding influences actin assembly kinetics. This system may translate to other cytoskeletal systems, as our results suggest that the alterations in filament assembly kinetics depend on the type of crowding molecules. A previous work has already investigated the effects of osmolytes and macromolecular crowding on tubulin assembly [51]. In our experiments, we kept other intracellular factors, such as pH, salt concentration, and temperature, constant because those can modulate actin assembly. For example, changes in solution pH influence actin polymerization, specifically, decreasing the pH to slightly acidic conditions results in enhanced filament elongation [42] and decreases the critical concentration of actin [52]. Overall, our in vitro experiments show how actin filament assembly in crowded environments is dependent on the type of crowding molecules in solution.

### 3.2. Crowding Modulates the Diffusion of Actin Subunits near Filament Barbed Ends

**Figure 4:**
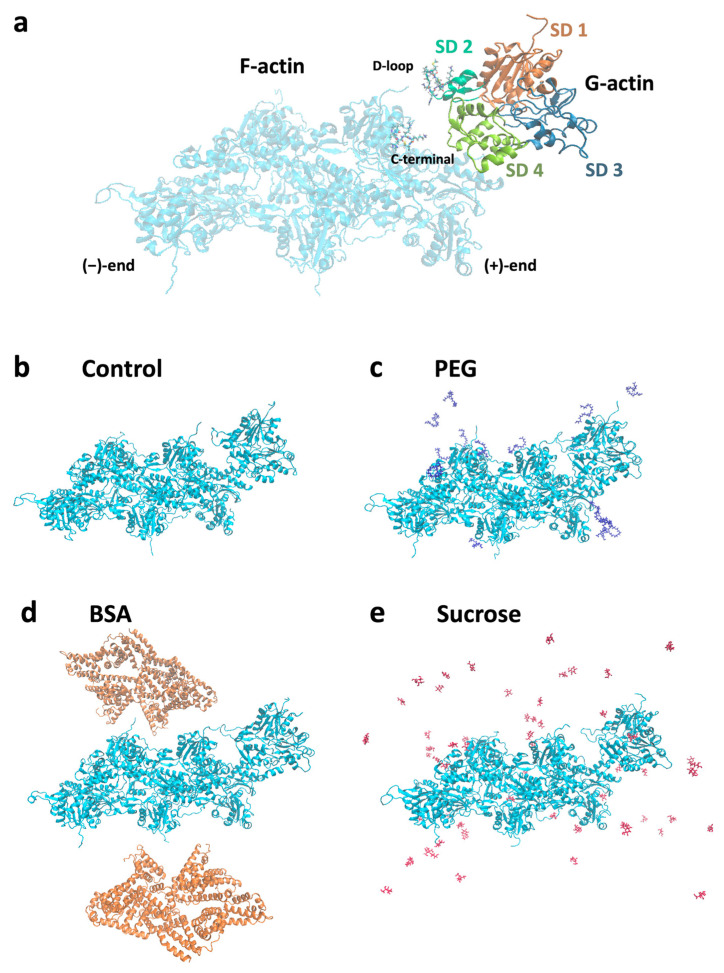
Molecular models for MD simulations of actin monomer (G-actin) and filament (F-actin) in the absence or presence of crowding molecules. (**a**) Actin subunit located near the barbed (+) end of the actin filament. The DNase I binding loop (D-loop) in actin subdomain (SD) 2 interacts with the C-terminal of actin filament. The D-loop was shown as a bond representation. Structure snapshots of actin with and without crowding agents after 5 ns of MD simulations. (**b**) Actin filament and subunit without crowders (control). (**c**–**e**) Actin filament and subunit in the presence of (**c**) polyethylene glycol (PEG), (**d**) bovine serum albumin (BSA), and (**e**) sucrose.

To better understand crowding-dependent actin polymerization at the molecular level, we performed explicit MD simulations to assess interactions between preformed filaments and subunits in various crowded conditions. MD simulations allowed us to analyze the conformations of an actin subunit initially located near the barbed end of a preformed filament (Figure 4). After 5 ns MD simulations, we observed that interactions between the actin subunit and filament changed with crowders. The DNase I binding loop (D-loop) is crucial for actin polymerization and stability of actin filaments. In the absence of crowding molecules, the D-loop of actin subdomain (SD) 2 did not interact significantly with the barbed end of the filament (Figure 4a,b). However, both PEG and BSA induced tight interactions between SD 2 and SD 4 of the subunit and the filament barbed end (Figure 4c,d). In contrast, only part of SD 2 of the actin subunit interacted with the filament barbed end with sucrose molecules (Figure 4e).

**Figure 5:**
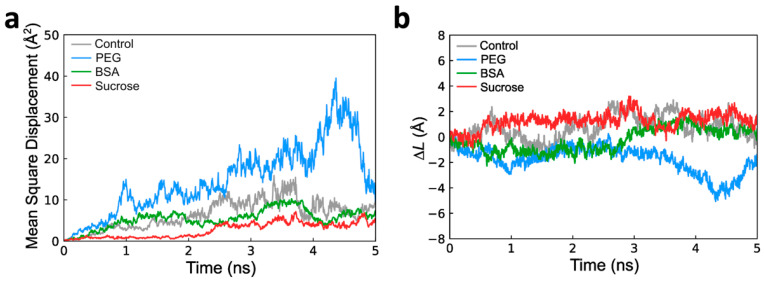
Dynamic characteristics of G-actin and inter-distance analysis between F- and G-actin during 5 ns molecular dynamics (MD) simulations. The mean square displacement (MSD) of actin subunits near filament barbed end was calculated after 5 ns MD simulations. (**a**) MSD of G-actin in the absence (control) and presence of crowding molecules (PEG, BSA, and sucrose). (**b**) The delta inter-distance (Δ*L*) between F- and G-actin was calculated by subtracting the varied inter-distance as a function of simulation time to initial inter-distance.

We further characterized the dynamic characteristics of actin subunits by calculating the mean square displacement (MSD) of a subunit near the filament barbed end and inter-distance variations between the filament and subunit (Figure 5). MSD analysis is an efficient way to evaluate molecular behaviors, such as protein–protein interactions and diffusion kinetics [48,53]. Measuring the MSD of an actin subunit near a filament allowed us to determine how crowding molecules influence actin subunit positioning at the end of the simulation compared to the subunit’s initial position. The MSD of an actin subunit can be correlated with filament assembly rates because the MSDs of the filaments were comparable to each other during our simulation time (5 ns). PEG induced higher MSDs of subunits than the control, indicating faster association of the actin subunit with the filament barbed end (Figure 5a). In contrast, sucrose resulted in lower MSDs of subunits compared to the control, suggesting slower actin polymerization (Figure 5a). In the presence of BSA, the MSD values were similar to the control. The diffusion coefficients of actin subunits obtained from the MSD calculations may evaluate the effects of crowding molecules on actin polymerization. The diffusion coefficient (*D*) of control G-actin (without crowding) was calculated to be 5.36 μm^2^/s (Table 1), which is consistent with reported values for actin monomer diffusion in cytoplasm and during cell protrusion [54,55]. PEG resulted in ~2.3 fold increase in the diffusion coefficient of G-actin when compared to the control, while sucrose decreased the diffusion coefficient (Table 1). Here, we noted the relatively higher diffusion coefficient of actin subunits with BSA compared to sucrose because the MSD of actin subunits with BSA was comparable to that of the control (Figure 5a and Table 1). In contrast to G-actin, the diffusion coefficients of actin filaments with or without crowding were comparable to each other (Supplementary Table S2).

To support the actin subunit diffusion analysis, we measured inter-distance variations (Δ*L*) between the center of mass of actin subunits and preformed filaments in the absence and presence of crowders. Inter-distance variation analysis can determine which direction the actin subunit is moving toward with relation to the actin filament in each of our simulated crowded environments. This analysis was necessary because there is a possibility that the actin subunit that moves away from the filament would also contribute to increases in the MSD and diffusion coefficients. During the MD simulations, PEG and BSA decreased the inter-distance variations between actin subunits and filament barbed ends from the initial time trajectory compared to the control, while sucrose increased the inter-distance between actin subunits and filament barbed ends (Figure 5b).

The kinetic features of actin subunits, such as the diffusion coefficient, have been identified for filament polymerization through experimental and theoretical approaches [14,17,56,57]. Recent computational studies have demonstrated that the MSD of target proteins in highly crowded environments determines kinetic and dynamic behaviors [58,59,60]. From our results, high MSDs affected by the presence of PEG implicated fast filament polymerization compared to the control, while low MSDs influenced by the presence of sucrose indicated that there was retardation of filament polymerization. The delta inter-distance between filaments and subunits can supplement the MSDs and diffusion coefficients of actin subunits, depending on the kind of crowding molecule. These calculated MSDs, inter-distances, and diffusion coefficients overall are consistent with experimentally observed filament elongation changes and actin polymerization rates, as shown in Figure 2 and Figure 3. Of note, while the sucrose and BSA molecules in the MD simulations were close to the experimental conditions, we were limited in the choice of available PEG molecular models, which restricted us to use a smaller size of PEG for our simulations.

### 3.3. Crowding Affects Filament Electrostatic Energy

Polar solvation free energy is a fundamental thermodynamic property that can be related to actin polymerization [56,61,62]. To evaluate actin polymerization in the presence of crowders, we measured the polar solvation free energies between preformed filaments and subunits from the 5 ns of MD simulation (Table 2). Our calculated polar solvation energy indicated that BSA and PEG have lower magnitudes of electrostatic energy, while sucrose displayed higher electrostatic energy compared to the control’s electrostatic solvation free energy (Table 2). The negative net charge (−27 e) of BSA may influence the lower polar solvation energy (up to 30× lower) observed when compared to the control, PEG, and sucrose. The lower polar solvation free energies of BSA and PEG implicated faster actin polymerization compared to the control, while the higher polar solvation energy value observed for sucrose suggests delayed F-actin polymerization. The charge distributions of molecular crowders calculated using the Adaptive Poisson–Boltzmann Solver (ABPS) indicated that either positive or negative charges were evenly distributed on PEG and BSA, while sucrose showed uneven charge distribution (Supplementary Figure S1). Differences in surface charge distributions on crowders may influence the polar solvation energy, as well as actin filament polymerization kinetics.

The electrostatic interactions between actin subunits and filaments modulate filament assembly [26,57,61,62]. A recent computational study demonstrated that electrostatic energy influences actin polymerization with respect to different salt concentrations [62]. Although the net charge of a crowding agent is different from the salt concentration, we can estimate that differences in polar solvation energies with respect to crowders are based on the charge distributions of crowding molecules. Since BSA and PEG have a greater hydrodynamic radius than sucrose, negative charges can be more evenly distributed in both PEG and BSA, compared to sucrose. The correlation between the size and charge distribution of crowders may determine the polar solvation energies. Our computational analysis suggests that electrostatic interactions between actin filaments and subunits influence actin polymerization in the presence of molecular and protein crowders.

## 4. Conclusions

In summary, we have demonstrated how solution crowding modulates actin polymerization kinetics through experimental and computational analyses. Direct visualization of actin filament assembly revealed the effects of various solution crowding on individual filament elongation rates. MD simulations showed that crowding molecules modulate the diffusion coefficients of actin monomers near filament barbed ends during polymerization. Our results suggest that alterations in actin monomer diffusion and electrostatic interactions between crowding agents and actin may be key factors in controlling filament assembly. Together, these results help us better understand how solution crowding can regulate actin assembly and cytoskeletal dynamics in vivo. Future studies can explore how crowding, with changes to other intracellular factors, including pH, ionic concentrations, and temperature, modulates actin assembly to more closely emulate the crowded conditions found within living cells.

## Figures and Tables

**Table 1:** Diffusion coefficient (*D*) of actin subunits without or with crowders. (Unit: μm^2^/s).

Control	PEG	BSA	Sucrose
5.36	12.4	4.55	2.25

**Table 2:** Polar solvation energy (Δ*G*) between actin filaments and actin subunits without (control) or with crowding molecules. (Unit: kcal/mol).

Control	PEG	BSA	Sucrose
−1295.6	−1373.08	−34,752.64	−1249.28

## Data Availability

Not applicable.

## Supplementary Materials

The following supporting information can be downloaded at https://www.mdpi.com/article/10.3390/biom13050786/s1. Figure S1: Charge distribution on (a) PEG, (b) sucrose, and (c) BSA; Table S1: Filament elongation rates quantified from TIRF imaging and relative assembly rates (compared to control) from TIRF analysis and pyrene fluorescence assay; Table S2: Diffusion coefficient (*D*) of actin filament without or with crowders; Video S1: Control actin filament elongation over time; Video S2: Actin filament elongation over time in 1% *w*/*w* PEG crowded conditions; Video S3: Actin filament elongation over time in 3% *w*/*w* PEG crowded conditions; Video S4: Actin filament elongation over time in 5% *w*/*w* PEG crowded conditions; Video S5: Actin filament elongation over time in 5% *w*/*w* BSA crowded conditions; Video S6: Actin filament elongation over time in 10% *w*/*w* BSA crowded conditions; Video S7: Filament elongation over time in 15% *w*/*w* BSA crowded conditions; Video S8: Filament elongation over time in 5% *w*/*w* sucrose crowded conditions; Video S9: Filament elongation over time in 10% *w*/*w* sucrose crowded conditions; Video S10: Filament elongation over time in 15% *w*/*w* sucrose crowded conditions.
