# Supplementary material for: Molecular Basis for Actin Polymerization Kinetics Modulated by Solution Crowding
Source: Biomolecules. 2023 May 2;13(5):786. doi: 10.3390/biom13050786 (PMC10216264; doi:10.3390/biom13050786)
Supplement: Supplementary file 1 [file biomolecules-13-00786-s001.zip › biomolecules-2293127-supplementary.pdf]

Supplementary Materials

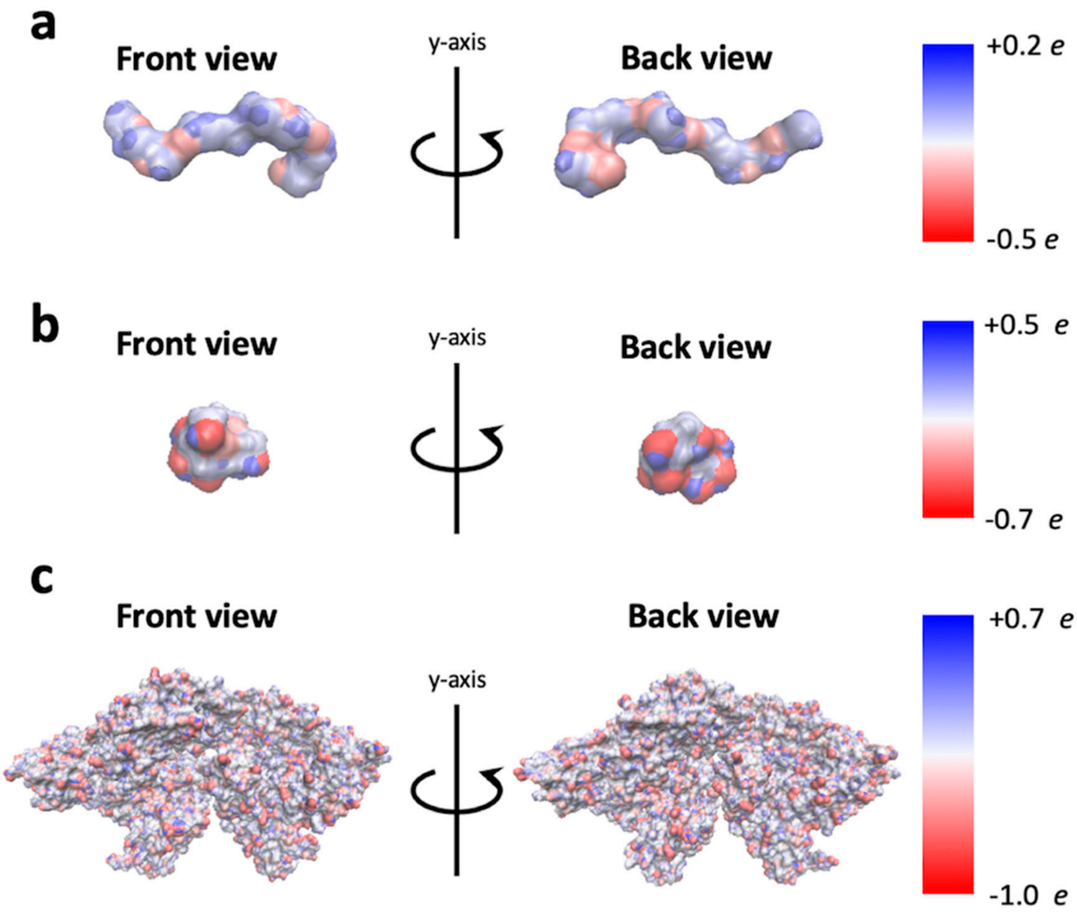

Figure S1. Charge distribution on (a) PEG, (b) sucrose, and (c) BSA. To describe the charge distribution of each crowder, we represented the front and back view of each crowder. Charge distribution of each crowder was obtained using the Adaptive Poisson-Boltzmann Solver (APBS) plugin of Visual Molecular Dynamics 1.9.1. (VMD).

Table S1. Filament elongation rates quantified from TIRF imaging and relative assembly rates (compared to control) from TIRF analysis and pyrene fluorescence assay.

Condition	Filament elongation rate (nm/s) (average ± s.e.m.)	Relative assembly rate from TIRF analysis	Relative polymerization rate from pyrene fluorescence assay
Control	35.9 ± 1.2	1.00	1.00
1% w/w PEG	50.0 ± 1.5	1.39	1.44
3% w/w PEG	61.3 ± 1.5	1.71	1.37
5% w/w PEG	51.2 ± 1.9	1.43	1.32
5% w/w BSA	42.0 ± 2.0	1.17	0.89
10% w/w BSA	37.8 ± 2.1	1.05	0.60
15% w/w BSA	38.1 ± 2.4	1.06	0.46
5% w/w Sucrose	30.5 ± 0.8	0.85	0.86
10% w/w Sucrose	23.9 ± 0.8	0.67	0.80
15% w/w Sucrose	18.9 ± 0.6	0.53	0.69

Table S2. Diffusion coefficient (*D*) of actin filament without or with crowders. (Unit: $\mu m^2/s$)

Control	PEG	BSA	Sucrose
1.62	1.90	1.97	1.74

Video S1: Control actin filament elongation over time. Actin was flowed into functionalized flow cells in the presence of 1X KMI buffer (10 mM imidazole, pH 7.0, 50 mM KCl, 2 mM $MgCl_2$, 0.2 mM $CaCl_2$, 1 mM ATP, and 1 mM DTT). Time stamp is in min:sec; scale bars, 5 μm.

Video S2: Actin filament elongation over time in 1% w/w PEG crowded conditions (P1%). Actin was flowed into functionalized flow cells in the presence of 1X KMI buffer containing 1% w/w PEG. Time stamp is in min:sec; scale bars, 5 μm.

Video S3: Actin filament elongation over time in 3% w/w PEG crowded conditions (P3%). Actin was flowed into functionalized flow cells in the presence of 1X KMI buffer containing 3% w/w PEG. Time stamp is in min:sec; scale bars, 5 μm.

Video S4: Actin filament elongation over time in 5% w/w PEG crowded conditions (P5%). Actin was flowed into functionalized flow cells in the presence of 1X KMI buffer containing 5% w/w PEG. Time stamp is in min:sec; scale bars, 5 μm.

Video S5: Actin filament elongation over time in 5% w/w BSA crowded conditions (B5%). Actin was flowed into functionalized flow cells in the presence of 1X KMI buffer containing 5% w/w BSA. Time stamp is in min:sec; scale bars, 5 μm.

Video S6: Actin filament elongation over time in 10% w/w BSA crowded conditions (B10%). Actin was flowed into functionalized flow cells in the presence of 1X KMI buffer containing 10% w/w BSA. Time stamp is in min:sec; scale bars, 5 μm.

Video S7: Actin filament elongation over time in 15% w/w BSA crowded conditions (B15%). Actin was flowed into functionalized flow cells in the presence of 1X KMI buffer containing 15% w/w BSA. Time stamp is in min:sec; scale bars, 5 μm.

Video S8: Actin filament elongation over time in 5% w/w sucrose crowded conditions (S5%). Actin was flowed into functionalized flow cells in the presence of 1X KMI buffer containing 5% w/w sucrose. Time stamp is in min:sec; scale bars, 5 μm.

Video S9: Actin filament elongation over time in 10% w/w sucrose crowded conditions (S10%). Actin was flowed into functionalized flow cells in the presence of 1X KMI buffer containing 10% w/w sucrose. Time stamp is in min:sec; scale bars, 5 μm.

Video S10: Actin filament elongation over time in 15% w/w sucrose crowded conditions (S15%). Actin was flowed into functionalized flow cells in the presence of 1X KMI buffer containing 15% w/w sucrose. Time stamp is in min:sec; scale bars, 5 μm.
